# Plasticity of the Root System Architecture and Leaf Gas Exchange Parameters Are Important for Maintaining Bottle Gourd Responses under Water Deficit

**DOI:** 10.3390/plants9121697

**Published:** 2020-12-03

**Authors:** Dinoclaudio Zacarias Rafael, Osvin Arriagada, Guillermo Toro, Jacob Mashilo, Freddy Mora-Poblete, Rodrigo Iván Contreras-Soto

**Affiliations:** 1 Institute of Biological Science, University of Talca, Talca 3460000, Chile; mosembaba@gmail.com (D.Z.R.); morapoblete@gmail.com (F.M.-P.); 2 Departamento de Ciencias Vegetales, Facultad de Agronomía e Ingeniería Forestal, Pontificia Universidad Católica de Chile, Santiago 306-22, Chile; arriagada.lagos.o@gmail.com; 3 Plant Stress Physiology Laboratory, Centro de Estudios Avanzados en Fruticultura (CEAF), Rengo 2940000, Chile; gtoro@ceaf.cl; 4 Limpopo Department of Agriculture and Rural, Bela-Bela 0480, South Africa; jacobmashilo@yahoo.com; 5 Instituto de Ciencias Agroalimentarias, Animales y Ambientales, Universidad de O’Higgins, San Fernando 3070000, Chile

**Keywords:** rhizoboxes, gaseous exchange, sub-Saharan Africa, root length density

## Abstract

The evaluation of root system architecture (RSA) development and the physiological responses of crop plants grown under water-limited conditions are of great importance. The purpose of this study was to examine the short-term variation of the morphological and physiological plasticity of *Lagenaria siceraria* genotypes under water deficit, evaluating the changes in the relationship between the root system architecture and leaf physiological responses. Bottle gourd genotypes were grown in rhizoboxes under well-watered and water deficit conditions. Significant genotype-water regime interactions were observed for several RSA traits and physiological parameters. Biplot analyses confirmed that the drought-tolerant genotypes (BG-48 and GC) showed a high net CO_2_ assimilation rate, stomatal conductance, transpiration rates with a smaller length, and a reduced root length density of second-order lateral roots, whereas the genotypes BG-67 and Osorno were identified as drought-sensitive and showed greater values for average root length and the density of second-order lateral roots. Consequently, a reduced length and density of lateral roots in bottle gourd should constitute a response to water deficit. The root traits studied here can be used to evaluate bottle gourd performance under novel water management strategies and as criteria for breeding selection.

## 1. Introduction

Drought is widely recognized as one of the most significant agricultural constraints in many regions worldwide, accounting for more than 80% of crop damage and losses [1]. In Mediterranean regions, for instance, the increase in annual average temperatures and the lower-than-average precipitation affect food production and sustainability in various agricultural systems [2]. In the context of climate change, it is highly probable that drought stress intensity will increase in the future as a result of more variable and unpredictable precipitation patterns. In the Mediterranean-like climate of central Chile, which is the main region for fruit and vegetable production in Chile, this phenomenon could potentially induce economic losses in agricultural systems. In fact, a recent study has indicated that Central Chile will likely experience detrimental effects on water availability and vegetation changes that will have social and economic impacts [3].

Chile is one of the major contributors to fruit and vegetable production in South America. In addition, Central Chile plays an important role and has positioned itself as a leading exporter of diverse agricultural products. Vegetable crop production in this region is dominated by small-scale farmers whose lands are vulnerable to climate change [4,5]. The increasing probability of drought occurrences coupled with the increasing demand for food for the growing human population indicate the need to develop crop management strategies that improve water-use efficiency and productivity and increase crop yield outputs, especially under water-restricted agricultural systems [6].

Drought tolerance in plants is associated with the modification of various morphological and physiological responses. These responses improve the adaptation and production of crops grown under water-limited conditions. The most common physiological parameters associated with drought tolerance in the short-term include enhanced net CO_2_ assimilation by the control of stomatal conductance and reduced transpiration rates for water conservation [7]. The maintenance of these physiological responses is widely associated with sustainable crop production in water-stressed environments [8]. Among the various plant organs, root development/morphology plays an important role associated with water-extraction from the soil profile, especially when water is limited [9,10]. The root system has great potential for improving plant adaptation and production under drought stress conditions [10,11,12]. In this context, Lynch [6] proposed that reduced root development would be advantageous for drought resistance in high-input agroecosystems. Root traits that improve water capture include fewer axial roots, a reduced density of lateral roots, and a greater loss of roots that do not contribute to water capture [6].

Several studies have reported a significant correlation between root and shoot traits, suggesting a coordinated strategy between below- and above-ground plant organs in response to water deficit [10,13]. These findings have enabled the selection of both root and shoot traits to improve drought tolerance and increase yield potential in several plant species, such as common bean [14], tomato [15], and quercus [16]. Moreover, Hund et al. [17] found that tolerant maize genotypes developed longer crown roots, which increased transpiration, stomatal conductance, and relative water content. Another study, also involving maize cultivars, supported the assumption that water stress reduces the production of crown roots, and lines with fewer crown roots had substantially deeper rooting and a greater capture rate of subsoil water and, consequently, improved the plant water status, stomatal conductance, leaf and canopy photosynthesis, biomass, and seed yield [18]. These results indicate that both root and physiological traits confer drought adaptation and should be useful for screening and selection for breeding purposes.

Bottle gourd (*Lagenaria siceraria* (Mol. Standl)) is an important cucurbit crop that is often grown under rainfed conditions in arid and semi-arid ecosystems. In semi-arid regions of sub-Saharan Africa, for instance, genetically diverse landraces of bottle gourd are commonly cultivated by local farmers in water-restricted conditions, yielding reasonable fruit production as a consequence of several years of selection and cultivation [8,19]. In this sense, the investigation of wild species or landraces from different gene pools could be useful to identify the morpho-physiological traits related to drought tolerance [20]. In addition, in genotypes of South African bottle gourd, Mashilo et al. [8] found that enhanced instantaneous water-use and intrinsic water-use efficiencies linked to high net CO_2_ assimilation (An), stomatal conductance (gs), and transpiration (E) rates were significantly associated with drought tolerance. In the present study, we hypothesized that, in the initial development of bottle gourd, enhanced physiological performance could be associated with changes in root phenes due to water reduction. In fact, there is a lack of information regarding the relationships that may exist between root system architecture (RSA) traits and physiological responses in bottle gourd. In light of this, the objective of this study was to examine the short-term variation of the morphological and physiological plasticity of *Lagenaria siceraria* genotypes under water deficit, evaluating the changes in the relationship between the root system architecture and leaf physiological responses.

## 2. Results

### 2.1. Differences in Water Consumption of Bottle Gourd Genotypes

**Figure 1:**
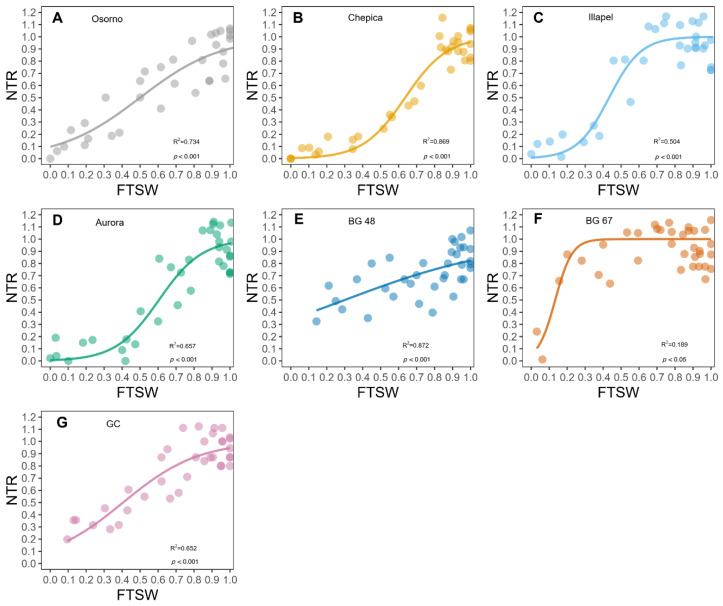
Normalized transpiration (NTR) response to fractions of transpirable soil water (FTSW) of seven genotypes of bottle gourd. Segmented regression indicated a threshold FTSW value above which there was a linear plateau of ~1.0 and below which there was a linear decline of NTR in response to decreasing FTSW. The genotypes are named on every figure.

The plot of the normalized transpiration of bottle gourd genotypes against the fraction of transpirable soil water (FTSW) is shown in Figure 1. In most of the genotypes, except for BG-48 (Figure 1E), a relatively high FTSW was observed with normalized transpiration (NTR) values of ~1. Illapel and BG-67 decreased the FTSW below a critical threshold value, and there was a marked linear decrease in NTR in response to further declines in FTSW. Segmented regression indicated that the threshold value for transpiration occurred at an FTSW that ranged from 0.80 (±0.1) for Chepica to an FTSW of 0.37 (±0.03) for BG-67 (Figure 1, Table 1). Osorno, Chepica, Aurora, and BG-48 genotypes showed high FTSW threshold values of 0.77, 0.80, 0.76, and 0.82, respectively, compared with the relatively low FTSW threshold values recorded for Illapel (0.47) and BG-67 (0.37).

### 2.2. Analysis of Variance and Mean Comparison for Physiological Parameters, Biomass, and Root System Architecture Traits

An analysis of variance (ANOVA) showed a highly significant (*p* < 0.001) effect of the genotype-water regime interaction for only stomatal conductance (gs), transpiration rate (E), and intrinsic water-use efficiency (WUEi) (Table 2). In most of the tested bottle gourd genotypes, the contrasting means in the comparison of the physiological traits under both water regimes showed that water deficit significantly reduced all traits (Tables S2–S4). In addition, BG-48 and GC genotypes showed significant differences between well-watered (WW) and water deficit (WD) treatments for stomatal conductance (gs) and transpiration rate (E) (Tables S2 and S3). On the other hand, for WUEi, the same genotypes showed an increment under the WD condition, whereas non-significant differences were observed for WUEins (Tables S4 and S5). For the same physiological traits, other genotypes showed a reduction in WUEi under the WD condition (Table S4). For RSA parameters, the genotype-water regime interaction effect was non-significant for the average root length of tap and basal roots (ARL), root angle of the first-order lateral of the tap and basal roots (ARA_1_), and root length density of the lateral of the tap and basal roots (RLD_L_). However, RSA traits measured in first-order and second-order lateral tap and basal roots—ARL_1_, ARL_2_, ARA_2_, and RLD_L1_—were influenced by the genotype-water regime interaction (Table 2). BG-48 showed a significant reduction in the length of lateral roots (i.e., ARL_1_ and ARL_2_) and a reduced density of lateral roots. Contrastingly, GC also showed a significant increment for both the length and density of lateral roots. Non-significant differences were observed for the genotypes Osorno, Chepica, and BG-67 for the same RSA traits (Table 3).

### 2.3. Correlations between Physiological and Root System Architecture Traits under Well-Watered and Water Deficit Conditions

**Figure 2:**
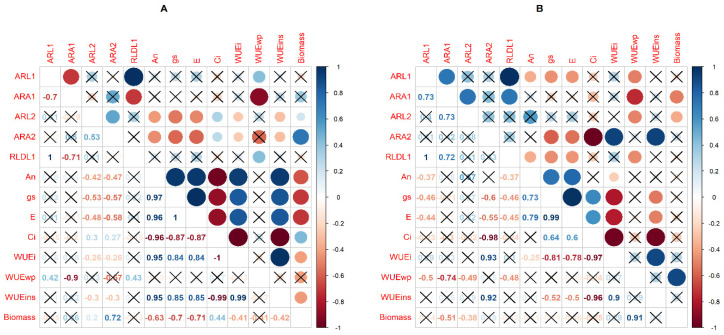
Pearson correlation coefficients among physiological and root system architecture (RSA) traits assessed in genotypes of bottle gourd in water deficit (**A**) and well-watered (**B**) conditions. Net CO_2_ assimilation rate (An); stomatal conductance (gs); intercellular CO_2_ concentration (Ci); transpiration rate (E); instantaneous water-use efficiency (WUEins); intrinsic water-use efficiency (WUEi); and whole plant water use efficiency (WUEwp). Average root length of the first-order lateral of the tap and basal roots (ARL_1_) and second-order lateral of the tap and basal roots (ARL_2_); average root angle of the first-order lateral of the tap and basal roots (ARA_1_) and second-order lateral of the tap and basal roots (ARA_2_); and root length density of the first-order lateral of the tap and basal roots (RLD_L1_). Positive correlations are displayed in blue and negative correlations in red. The color intensity and the size of the circle are proportional to the correlation coefficients. On the right side of the correlogram, the color legend shows the correlation coefficients and the corresponding colors.

Pearson correlation coefficients between physiological and RSA traits among the evaluated bottle gourd landraces under WW and WD conditions are presented in Figure 2. Negative and significant associations were observed for several physiological and RSA traits and biomass production under water deficit conditions. The net CO_2_ assimilation (An), stomatal conductance (gs), and transpiration rate (E) were negatively and significantly correlated with ARL_2_, ARA_2_, and biomass. On the other hand, Ci values were positively and significantly correlated with ARL_2_, ARA_2_, and biomass, but negatively correlated with leaf gas exchange parameters (An, gs, and E). Intrinsic and instantaneous water use-efficiencies were negatively and significantly correlated with ARL_2_, whereas WUEwp was negatively and significantly correlated with ARA_2_, but positively correlated with ARL_1_ and RLD_L1_.

Under the WW condition, biomass was negatively correlated with some RSA traits (ARA_1_ and ARL_2_) and positively correlated with WUEwp. ARA_2_ was negatively and significantly correlated with gs and E and positively correlated with WUEi and WUEins. WUEins and WUEi were both negatively correlated with gs, E, and Ci. Furthermore, WUEwp was negatively correlated with RSA traits (ARL_1_, ARA_1_, and ARL_2_) (Figure 2).

### 2.4. Principal Component Analysis for the Differentiation of Drought-Tolerant and Sensitive Bottle Gourd Genotypes

Principal component analyses of physiological and RSA parameters measured under water deficit and well-watered conditions are presented in Table 4. Under the WD condition, the total variability of the three-dimensional space was efficiently summarized by the two principal components, which accounted for 51% and 26% of the variability, respectively. The first component consisted of high positive loadings for leaf gas exchange parameters as well as An, gs, E, WUEi, and WUEins and negative loadings for some RSA traits (ARA_2_, ARA_2_, and ARL_2_), biomass, and Ci. In contrast, the second component consisted of high positive and negative loadings of root traits such as RLD_L1_, ARL_1_, ARA_1_, ARL_2_, and ARA_2_. Under the WW condition, the first component consisted of negative loadings of RSA traits and biomass, while the leaf gas exchange parameters consisted of positive loadings (An, gs, E, and Ci) that accounted for 43% of the total variation. On the other hand, the second component consisted of negative loadings for most of the leaf gas exchange parameters (An, gs, E, and Ci) and root traits (RLD_L1_, ARL_1_, ARA_2_, and ARL_2_), which accounted for 29% of the total variation.

**Figure 3:**
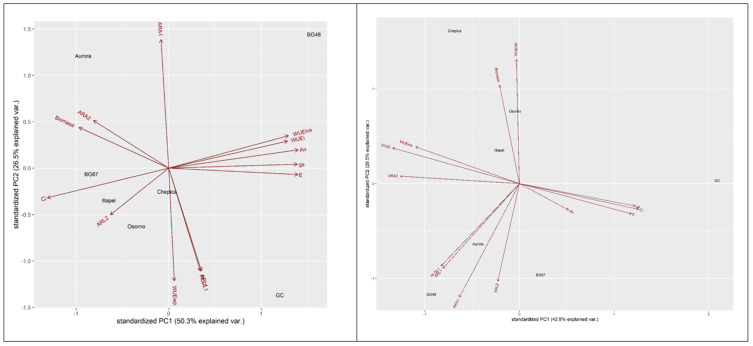
Principal component (PC) biplot showing the percentage of variance explained by PC1 and PC2, and grouping of bottle gourd genotypes based on physiological and root system architecture traits under water deficit and well-watered conditions.

A principal component biplot (PC1 and PC2) was used to visualize the relationships between bottle gourd genotypes based on physiological and RSA parameters (Figure 3). In this biplot, smaller angles with the same direction among the vectors represented the most informative and correlated physiological and/or root traits, identifying groups of genotypes based on the assessed traits. The genotypes that were closed or in the same direction as the vectors were plotted as associated with an increase or reduction of these traits. Under the WW condition, genotypes Aurora and BG-48 were grouped with high values of ARA_1_, ARL_1_, ARL_2_, and RLD_L1_. Osorno and Illapel were differentiated by high values of WUEwp. On the other hand, reduced values of the leaf gas exchange parameters of An, gs, E, and Ci were associated with BG-67 and GC. Under the WD condition, Aurora was grouped with high values of ARA_1_. Osorno, Illapel, and BG-67 were grouped as expressing high ARA_2_, ARL_2_, and Ci. On the contrary, BG-48 was differentiated by high An, gs, E, WUEins, and WUEi values and a reduction in the length and density of lateral roots (ARL_1_, ARL_1_, and RLD_L1_). GC possessed high Ci, gs, and E (Figure 3).

### 2.5. Morphological and Physiological Plasticity

**Figure 4:**
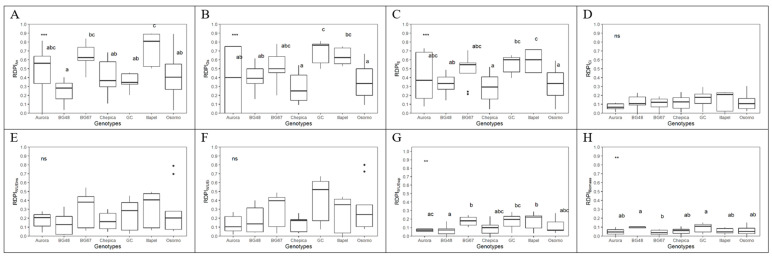
Relative distance plasticity index (RDPI) considering physiological traits (**A**–**G**) and biomass (**H**) in seven bottle gourd genotypes subjected to the water deficit condition. *p*-value for difference between genotypes (*p* < 0.001, Tukey’s test). Lowercase letters compare the RDPI in each genotype (Tukey test, *p* < 0.001). ns = non-significant analysis of variance (ANOVA); ** significant at 1%; *** significant at 0.1%.

**Figure 5:**
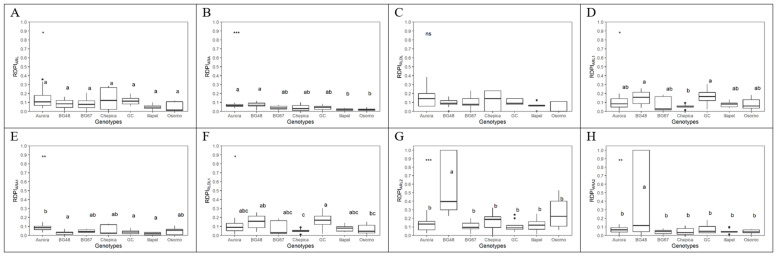
Relative distance plasticity index (RDPI) considering morphological traits (**A**–**H**) in seven bottle gourd genotypes subjected to the water deficit condition. *p*-value for difference between genotypes (*p* < 0.001, Tukey’s test). Lowercase letters compare the RDPI in each genotype (Tukey test, *p* < 0.001). ns = non-significant ANOVA; * significant at 5%; ** significant at 1%; *** significant at 0.1%.

Among the seven bottle gourd genotypes, significant differences were observed in the relative distance plasticity index (RDPI) in physiological and morphological traits (biomass and RSA) (Figure 4 and Figure 5). In general, low plasticity was observed for physiological and morphological traits. No significant differences were observed among genotypes for RDPI in Ci, WUEi, and WUEins. GC and Illapel showed higher RDPI values for leaf gas exchange (Gs and E) than Osorno and Chepica. Furthermore, GC and BG-48 showed higher RDPI values for biomass when compared with the other five genotypes (Figure 4). Regarding the root morphological traits, no significant differences were observed among genotypes for RDPI in ARL and RLD_L_. BG-48 showed higher plasticity for the second-order lateral of the tap and basal roots (ARA_2_ and ARL_2_) and the root length density of the first-order lateral of the tap and basal roots when compared with the other six genotypes (Figure 5).

## 3. Discussion

When studying plant responses to water deficit, several morphological and physiological traits have been evaluated and reported [20,21]. Such a large dataset of numbers and variables makes it difficult to form an overall idea of how water deficit affects plants and how plants respond to such a limiting condition [22]. The approach of the relative distance plasticity index used here has been used to study plant adaptations under different conditions or environments and to evaluate growth responses under stressful conditions [22,23,24]. Our results for the RDPI showed that most of the traits studied showed some level of plasticity in response to water reduction, even though the plasticity presented here was relatively low (RDPI < 0.5); in addition, there was also some evidence that not all traits that contributed significantly and highly to variation presented higher plasticity indices in response to water reduction.

In the present study, the root morphological and physiological plasticity of drought-related traits and the negative correlation between leaf gas exchange parameters with lateral tap and basal roots allowed us to characterize the response to water reduction of bottle gourd. In different plant species, previous studies have also reported traits that were responsible for plastic responses with the aim of obtaining an integrative index related to the sensitivity to drought stress of various genotypes [16,22,24,25]. For instance, in our study, high variability and a genotype-dependent relative plasticity index were observed between bottle gourd genotypes; in particular, the Osorno genotype showed the lowest physiological and morphological plasticity index, whereas Illapel and BG-48 showed higher physiological and morphological plasticity indices, respectively. Furthermore, these results confirmed that, in some bottle gourd genotypes, the leaf gas exchange parameters were positively influenced by drought stress [8,26] and, consequently, could be used as drought-related traits. On the other hand, this study also shows that conclusions regarding the response of bottle gourd to water reduction are a result of different strategies associated with root morphological drought-related traits.

Plant responses, soil water availability, and the water uptake capacity from shallow or deep soils have been widely studied as important key factors to assess the tolerance degree to water deficit of different plant species [27,28,29]. In general, two strategies have been described to explain the behavior of plants to face water deficit: a “productive” strategy, which attempts to maintain open stomata, assuming water losses, but increasing net CO_2_ assimilation to yield biomass; and a “conservative” strategy, which ensures water conservation in the soil and promotes early stomatal closure in response to water deficit [30]. In this study, we found a variability in the FTSW threshold between South African and Chilean genotypes, highlighting BG-48, Chépica, Osorno, Aurora, and GC genotypes as exhibiting “conservative” behavior, while Illapel and BG-67 showed “productive” behavior.

Some physiological traits (An, Gs, and E) revealed that Chepica and Osorno were more sensitive to water deficit than GC and Illapel. Specifically, Osorno, BG-67, and Chepica showed a severe reduction of some leaf gas exchange parameters (mean values of stomatal conductance, photosynthetic rate, and transpiration) as a result of water stress when compared with BG-48 and GC. BG-67 and Osorno genotypes recorded reductions of 91% and 84% in photosynthetic rates, 88% and 84% reductions in stomatal conductance, and 84% and 81% reductions in transpiration, respectively. Similarly, a reduction in stomatal conductance and the CO_2_ assimilation rate under water deficit has been reported in different plant species including watermelon [31], squash [32], and quercus [29]. In previous studies based on the physiological performance of *L. siceraria*, Mashilo et al. [8] classified BG-48 and GC as drought-tolerant genotypes. Our study also revealed contrasting abilities to tolerate water stress, where bottle gourd genotypes that originated from arid and semi-arid environments (i.e., BG-48 and GC) showed better tolerance compared with Chilean genotypes grown in temperate or cold environments (i.e., Osorno and Chepica).

The BG-48 and GC genotypes, which are tolerant to water deficit [8], recorded a decreased intercellular CO_2_ concentration due to water stress, although this tendency was not significant (Table S6). These findings may confirm that, under water deficit conditions, the stomatal closure reduces the internal CO_2_ concentration of the leaf, as proposed by Cornic [33], Zhang et al. [34], and Flexas et al. [35]. However, there are contradicting reports on the mechanism responsible for stomatal closure. Some studies endorse the view that chemical signals are responsible for stomatal closure, while others support the idea that hydraulic signals are responsible [7]. This report probably supported the contrasting results previously reported for bottle gourd by Mashilo et al. [8], which revealed that drought-stressed genotypes (BG-48 and GC) showed an increased intercellular CO_2_ concentration irrespective of reduced stomatal conductance, photosynthetic, and transpiration rates. Although similar results that reported increased CO_2_ concentration were observed under water stress in cowpea [36], maize [37], and wheat [38], we suggest that more research is necessary on stomatal closure as a response to water deficit in bottle gourd.

Regarding the morphological plasticity indices, BG-48 and GC genotypes presented higher plasticity than the other five genotypes, which was based on ARL_1_, ARL_2_, ARA_2_, RLD_L1_, and biomass. As BG-48 and GC genotypes presented a greater biomass than the other five genotypes, we may argue that the secondary growth and ability to maintain or increase root length and the density of lateral tap and basal roots under water deficit may be related to the good growth and yield performance of bottle gourd under drought conditions. It is important to note that ARL_2_ and ARA_2_ showed relatively moderate plasticity in comparison with the other RDPIs. In addition, under the water deficit condition, BG-48 had specific phene states as the reduced length (ARL_1_ and ARL_2_) and density of lateral tap and basal roots (RLD_L1_) permit greater resource allocation to deeper roots. In cassava and maize, some authors noted that genotypes with high yield potential under drought are characterized by having a more intensive and extensive fine root system, which enables the acquisition of more water from larger and deeper volumes of soil [24,39,40]. In fact, Lynch [6], in a revision of root phenotypes for drought resistance, proposed that specific root phenes such as fewer axial roots and a reduced density of lateral roots may contribute to improving water capture in dry topsoil.

In addition to the morpho-physiological plasticity index, principal component analysis was conducted to discriminate tolerant and susceptible bottle gourd genotypes based on their physiological and RSA traits. In particular, the PCA was able to reduce and group physiological and root morphological traits into components according to their ability to describe the variability among bottle gourd genotypes under the water deficit condition. Plotting the bottle gourd genotypes by means of their component scores, PC1 separated BG-48 with positive values of An, gs, E, WUEins, and WUEi and a reduction in the length and density of lateral roots (ARL_1_, ARL_2_, and RLD_L1_). This finding indicated that water reduction led to fewer axial roots and a reduced density of lateral roots, which may contribute to improving water capture in dry topsoil. On the other hand, the genotype BG-67 showed a reduction in leaf gas exchange parameters with some increment in the length of lateral roots, which may be considered another strategy associated with “productive” behavior.

## 4. Material and Methods

### 4.1. Plant Material

The plant material used in this study consisted of seven bottle gourd genotypes. Three were commercial varieties sourced from the Limpopo Department of Agriculture and Rural Development (Towoomba Research Station) of South Africa, one was a commercial variety from Chile, and the rest were accessions collected from three regions of Chile. Breeding varieties from South Africa were identified with a high level of drought tolerance and cultivated under dryland conditions with limited agricultural inputs (i.e., fertilization and irrigation) [8]. Details of the bottle gourd genotypes are shown in Table S7.

### 4.2. Experimental Design and Growing Conditions

**Figure 6:**
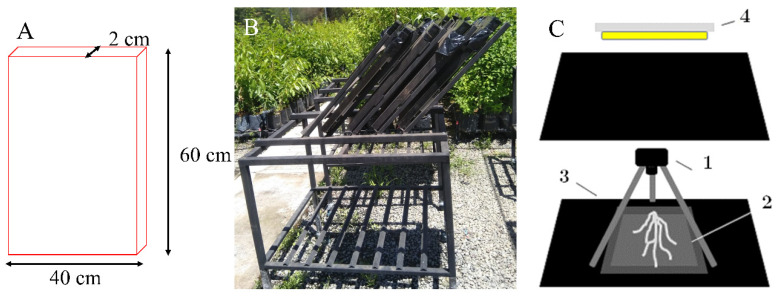
Rhizobox structure and dimensions used to evaluate root system architecture traits (**A**); angle and position of rhizobox in the field experiment (**B**); rhizobox (2) placed horizontally on a black surface (3) at a distance of ~134 cm from the digital camera (1) to avoid the effect of light (4) (**C**).

Bottle gourd seeds were sterilized by immersion in 2% (*v*/*v*) sodium hypochlorite in water for 10 min, rinsed twice with deionized water for 10 min, and germinated for 5–7 seven days at 20–25 °C in 7 cm × 7 cm × 8 cm (0.23 L) pots with peat and sand substrate in an equal ratio of 1:1. Plants with the first fully expanded true leaf and with an absence of damage or disease were considered as criteria for transplantation to the rhizobox. For root system architecture phenotyping, experiments were conducted in rhizoboxes (length × width × height = 60 × 2 × 40 cm), which were boxes with transparent plexiglass plates and covered by a non-transparent plastic box on the outside (Figure 6). Rhizoboxes were inclined by 45° to the horizontal plane with the plexiglass plate on the underside, so that roots could grow along the surface (Figure 6B). Each rhizobox was filled with ~2 kg of substrate (1:1 peat/sand *v*/*v*). Fertilizer was not applied during the entire experiment to avoid a confusion of the applied stress.

Bottle gourd plants were grown under field conditions, where the average air temperature was 23.8 ± 2.7 °C with a relative humidity of 54% and solar radiation level of 27 Mj/m^2^. The experiment was conducted in the 2019–2020 growing season in the field condition using a shade net cover (Raschel sun-shading net with 50% light transmittance). A completely randomized design with a 7 × 2 factorial arrangement and three replicates was used. Factors consisted of seven bottle gourd genotypes and two water regimes (well-watered and water deficit conditions).

### 4.3. Water Deficit Treatment, Fractions of Transpirable Soil Water, and Transpiration Rate

Twenty days after sprouting, the plants of each genotype were transplanted to the rhizobox. At this time, plants were subjected to two water availability irrigation conditions: well-watered (WW) and water deficit (WD). Plants under the WW condition were irrigated three times per week, adding water to reach the corresponding 100% of the substrate water content of each rhizobox during the period of the experiment (28 days). In contrast, the WD condition was induced by suspending the irrigation supply for 28 days, followed by weighting each rhizobox three times per week to determine the amount of water consumed by each plant for the assessed genotypes. The fraction of transpirable soil water (FTSW) relative to well-watered treatments, which represented the portion of remaining volumetric soil water available for transpiration on each day of the experiment, was used as the indicator of stress [41]. The FTSW for each day of the experiment was calculated using Equation (1):

FTSW = [Pot weight day n − Final pot weight]/[Initial pot weight − Final pot weight](1)

The normalized transpiration rates (NTRs) of WW and WD plants were determined by dividing the daily transpiration rate (gravimetrically) of each replication in each treatment of WD plants by the transpiration rate of WW plants. The NTR and FTSW were calculated for each rhizobox in the WD treatment using rhizobox weights recorded three times per week. For plants growing under the WD condition, the NTR of each bottle gourd genotype was plotted against the FTSW by fitting a segmented non-linear regression to determine the FTSW threshold value at which the NTR began to decline. The non-linear regression was fitted using R 4.0 [R Core Development Team, 2020].

### 4.4. Physiological Parameters and Biomass Production

Gas exchange parameters including the stomatal conductance (gs), transpiration rate (E), intercellular CO_2_ concentration (Ci), and net CO_2_ assimilation rate (An) were measured once per week for four weeks using a CIRAS-2 portable IRGA photosynthesis system (PPSystem, Hitchin, UK) with a controlled environment CIRAS PLC cuvette (broad windows 2.5 cm^2^). The CO_2_ concentration and photosynthetically active radiation inside the cuvette were adjusted to 400 µmol mol^−1^ and 1500 µmol m^−2^ s^−1^, respectively. The measurements were all carried out between 09:00 and 14:00 on clear days on the fifth and fully-expanded leaves of the plants. Intrinsic water-use efficiency (WUEi) was calculated as the ratio between An and gs, and instantaneous water-use efficiency (WUEins) as the ratio between An and E. To calculate the whole-plant water-use efficiency (WUEwp), three plants per genotype and water treatments were harvested at the end of the experiment. Leaves, shoots, and roots for each plant were separated and dried in an oven at 60 °C to obtain dry weights. The total biomass increase during the experiment was estimated as the difference between the whole-plant dry weights at the beginning and end of the experiment. Plant water consumed over the four-week period was estimated from the sum of the daily water consumption. WUEwp was determined according to Medrano et al. [42] using Equation (2):

(2) $${\mathrm{WUE}}_{\mathrm{wp}}\left({\mathrm{gL}}^{-1}\right)=\frac{\left(\mathrm{dry}\;\mathrm{weight}\;\mathrm{of}\;\mathrm{final}\;\mathrm{biomass}-\mathrm{dry}\;\mathrm{weight}\;\mathrm{of}\;\mathrm{initial}\;\mathrm{biomass}\right)}{\mathrm{total}\;\mathrm{water}\;\mathrm{consumed}}$$

Finally, to determine the dry-mass (biomass) of each genotype in WD and WW conditions, the stem and roots were put in an oven for a minimum of 48 h at 70 °C, and then the mass in grams was measured.

### 4.5. Root Parameters and Image Processing

To characterize the root system architecture (RSA) of plants grown under WW and WD conditions, the rhizoboxes were photographed once per week with a high-resolution Nikon digital camera (Nikon D3500) fitted with a Nikkor AF-P 18–55 mm 1:3.5–5.6 G lens. For standard imaging, the focus of the camera was placed vertically, which was also done to avoid the effect of light on the acrylic of the rhizoboxes. The rhizoboxes were placed horizontally on a black surface at a distance of ~134 cm from the camera to obtain the best focus of fine roots (Figure 6C). The focus of the camera was adjusted manually and remained fixed for all images of the rhizoboxes.

The CI-690 RootSnap was used to measure the root traits, and the RSA traits or root classes based on the site of origin were classified as proposed by Zobel and Waiser [43]. In our study, the tap and basal roots were used to calculate the average root length (ARL) and average root angle (ARA). Furthermore, the RSA was classified and measured as the average root length of the first-(ARL_1_) and second-order (ARL_2_) lateral tap and basal roots and the average root angle of the first-(ARA_1_) and second-order (ARA_2_) lateral tap and basal roots.

Other RSA parameters, including root length density (RLD), which was expressed as the total length of root per unit of volume of soil (RLD_L_), were calculated according to Johnson et al. [44] using Equation (3):

RLD_L_ = L/(A × D) (3)

where

- RLD_L_: root length density, based on the length of roots (cm root cm^3^ soil);
- L: total length of root observed under the rhizobox (cm);
- A: framework area observed in the rhizobox (60 × 40 = 2400 cm^2^);
- D: depth of the rhizobox (2 cm).

Two measurements of RLD_L_ were calculated as the RLD of tap and basal roots (RLD_L_) and the first-order lateral tap and basal roots (RLD_L1_).

### 4.6. Morphological and Physiological Plasticity Index

The relative distance plasticity index (RDPI) was calculated for morphological and physiological traits following Valladares et al. [23] and Marchiori et al. [22]. The data obtained at 28 days after transplanting were used to calculate the morphological and physiological plasticity, which indicated the relative phenotypic distance between individuals of the same genotype exposed to different treatments (WW and WD). Briefly, for each bottle gourd genotype, a 2 × 3 matrix of each morphological and physiological parameter was constructed, where the rows (*i*) represented the treatments and the columns represented the bottle gourd individuals (*j*) (i.e., the replicate for each treatment). We considered two water regimes (*i* = 1, 2) and three individuals of each bottle gourd genotype (*j* = 1, 2, 3). The phenotypic plasticity for a given variable *x* can be related to the difference of *x* between two individuals of the same genotype grown under different water treatments. The phenotypic plasticity was described by the absolute distance between two selected individuals (*j* and *j’*) of the same genotype grown under distinct water conditions (*i* and *i’*). Regarding this assumption for the whole data set, we computed pairwise distances across all individuals and water conditions. For a given variable *x*, the distance among values (d*ij→i’j’*) was the difference *xi’j’* − *xij*, and the relative distances (rd*ij→i’j’*) were defined as d*ij→i’j’*/(*xi’j’* + *xij*) for all pairs of individuals of a given genotype grown under different water availabilities. Finally, RDPI was calculated as ∑(rd*ij→i’j’*)/*n*, where *n* represented the number of distances. Detailed descriptions of the relative distance plasticity index and its bases are given in Valladares et al. [21] and Marchiori et al. [22].

The RDPI differences between genotypes were evaluated with a one-way ANOVA and post-hoc Tukey mean comparison test (*p* < 0.05) using the packages ‘ggpubr’, ‘plry’, and ‘multicompView’ and considering ‘bottle gourd genotypes’ as a factor.

### 4.7. Data Analysis

An analysis of variance (ANOVA) was performed after testing the homogeneity of variances and normality of the residuals using Bartlett and Shapiro–Wilk tests.

A two-way ANOVA was performed for physiological and RSA traits. For the multiple comparison analysis test, orthogonal contrasts were performed to compare the mean values of genotypes by the water regime interaction effect. Statistical analyses were performed using the PROC GLM procedure of SAS software (SAS version 9.3).

The mean values of the studied RSA traits and physiological parameters for each condition (WW and WD) were used to compute the Pearson’s linear correlation coefficients to describe the pattern of association between physiological and RSA traits in the R-package, using the “corrplot” function. Significance tests for the correlation coefficients were determined using Student’s *t*-test.

A principal component analysis (PCA) based on the correlation matrix was performed using the “princomp” function in R. The eigenvectors derived from the PCA were used to identify the variables that had a strong relationship with a specific principal component. The PC biplot was then generated using the “ggbiplot” package in R to describe and group bottle gourds for their level of drought tolerance according to Shah et al. [45].

## 5. Conclusions

In conclusion, our results provided evidence that most of the traits studied showed some level of plasticity in response to water reduction. Some RSA traits, such as a reduced length and density of lateral roots (RLD_L1_, ARL_2_, ARA_2_, and ARL_1_), were able to improve the morphological plasticity of root biomass production in bottle gourd under the water deficit condition. These findings may contribute to a better understanding of the drought-tolerant mechanisms conferred by root system architecture traits and the physiological responses of bottle gourd, leading to efficient selection criteria and enhancements of the drought adaptation and phenotypic plasticity in this vegetable crop.

## Figures and Tables

**Table 1:** Comparison of fractions of transpirable soil water threshold (FTSW_t_) values of seven genotypes of bottle gourd.

Genotype	FTSW_t_	S.E	*
BG-48	0.824	0.08	a
Chepica	0.800	0.02	b
Osorno	0.777	0.10	c
Aurora	0.760	0.04	c
GC	0.696	0.10	d
Illapel	0.474	0.02	e
BG-67	0.368	0.03	f

S.E: standard error; * lowercase letters compare FTSWt between genotypes (Tukey test, *p* < 0.05).

**Table 2:** Results of analysis of variance for physiological parameters and root system architecture traits evaluated in seven bottle gourd genotypes under well-watered and water deficit conditions.

Source of Variation	Significance (Physiological Traits)							
	An	gs	E	Ci	WUEi	WUEins	WUEwp	Biomass
Genotype (G)	**	**	**	ns	ns	ns	ns	ns
Water regime (W)	**	**	**	ns	ns	ns	**	**
G*W	ns	**	**	ns	**	ns	ns	ns
CV (%)	35.8	60.7	40.5	18.0	34.1	30.7	15.4	7.5
	**Significance (root system architecture traits)**							
	RLD_L_	ARL	ARA	RLD_L1_	ARL_1_	ARA_1_	ARL_2_	ARA_2_
Genotype (G)	**	**	**	ns	ns	**	ns	ns
Water regime (W)	ns	ns	ns	ns	ns	**	ns	ns
G*W	ns	ns	**	**	**	ns	**	**
CV (%)	16.2	16.2	5.3	14.9	14.9	7.2	19.8	7.5

CV (%): coefficient of variation in percentage; net CO_2_ assimilation rate (An), stomatal conductance (gs), transpiration rate (E), intercellular CO_2_ concentration (Ci), intrinsic water-use efficiency (WUEi), instantaneous water-use efficiency (WUEins), and whole plant water-use efficiency (WUEwp); average root length of tap and basal roots (ARL), root length of the first-order lateral of the tap and basal roots (ARL_1_), and root length of the second-order lateral of the tap and basal roots (ARL_2_); average root angle for the tap and basal roots (ARA), root angle of the first-order lateral of the tap and basal roots (ARA_1_), root angle of the second-order lateral of the tap and basal roots (ARA_2_), root length density of tap and basal roots (RLD), and root length density of the first-order lateral of the tap and basal roots (RLD_L1_). ns, non-significant; **, significant at 1% probability by the F-test, respectively.

**Table 3:** Results of orthogonal contrasting tests for the difference in mean values between water deficit (WD) and well-watered (WW) conditions for the average root length of the first-order and second-order of the lateral of tap and basal roots (ARL_1_ and ARL_2_), the average root angle of the second-order of the lateral of tap and basal roots (ARA_2_), and the root length density of the lateral tap and basal roots (RLD_L1_).

Genotype	ARL_1_ (cm)	ARL_2_ (cm)	RLD_L1_ (cm/cm^3^)	ARA_2_ (°)
Osorno	−27.9 ns	0.07 **	−0.01 ns	1.91 ns
Chepica	86.3 ns	2.5 ns	0.02 ns	3.04 ns
Illapel	2.85 ns	−0.27 ns	0 ns	−0.43 ns
Aurora	−62.5 ns	−4.83 ns	−0.01 ns	5.86 *
BG-48	44.1 **	−7.92 ***	0.01 *	1.11 *
BG-67	40.4 ns	−3.84 ns	0.01 ns	−1.06 ns
GC	−47.3 **	7.7 ns	−0.01 *	−0.01 ns

ns: non-significant; * significant at 5%; ** significant at 1%; *** significant at 0.1%.

**Table 4:** Principal component analysis showing eigenvectors, eigenvalues, and percentage of variance of physiological and root system architecture traits of seven bottle gourd genotypes under water deficit and well-watered conditions.

Traits	Water Deficit (Eigenvectors)			Well-Watered (Eigenvectors)		
	PC1	PC2	PC3	PC1	PC2	PC3
RLD_L1_	0.09	−0.42	0.38	−0.25	−0.32	−0.21
ARL_1_	0.09	−0.42	0.38	−0.24	−0.33	−0.22
ARA_1_	−0.02	0.53	0.12	−0.19	−0.44	0.09
ARL_2_	−0.17	−0.19	0.40	−0.07	−0.37	0.37
ARA_2_	−0.22	0.19	0.52	−0.38	0.03	0.28
An	0.39	0.08	0.05	0.16	−0.10	0.56
gs	0.38	0.02	−0.04	0.37	−0.08	0.27
E	0.39	−0.03	−0.01	0.35	−0.12	0.29
Ci	−0.36	−0.12	−0.19	0.37	−0.09	−0.26
WUEi	0.35	0.11	0.20	−0.40	0.14	0.11
WUEwp	0.01	−0.46	−0.24	−0.01	0.48	0.11
WUEins	0.36	0.13	0.17	−0.33	0.14	0.33
Biomass	−0.27	0.17	0.31	−0.06	0.38	0.03
Eigenvalues	2.56	1.86	1.35	2.36	1.92	1.57
Proportion of total variance (%)	0.50	0.26	0.14	0.43	0.29	0.19
Cumulative variance (%)	0.50	0.76	0.91	0.43	0.72	0.91

## Supplementary Materials

The following are available online at https://www.mdpi.com/2223-7747/9/12/1697/s1, Table S1: Increment and reduction of CO_2_ assimilation (An), stomatal conductance (gs), and intrinsic water-use efficiency (An/gs) between initial and final mean values (experimental time period) of stress treatments in seven bottle gourd landraces. Table S2: Results of contrast tests comparing the mean values difference between well-watered (WW) and water deficit (WD) conditions for stomatal conductance (gs). Table S3: Results of contrast test comparing the mean value differences between well-watered (WW) and water deficit (WD) conditions for transpiration (E). Table S4: Results of contrast tests comparing the mean value differences between well-watered (WW) and water deficit (WD) conditions for intrinsic water use efficiency (WUEi). Table S5: Results of contrast tests comparing the mean value differences between well-watered (WW) and water deficit (WD) conditions for instantaneous water use efficiency (WUEins). Table S6: Results of contrast tests comparing the mean value differences between well-watered (WW) and water deficit (WD) conditions for intercellular CO_2_ concentration (Ci). Table S7: Origin and geographical coordinates of seven bottle gourd genotypes evaluated under well-watered and water deficit conditions.
