# Supplementary material for: Plasticity of the Root System Architecture and Leaf Gas Exchange Parameters Are Important for Maintaining Bottle Gourd Responses under Water Deficit
Source: Plants (Basel). 2020 Dec 3;9(12):1697. doi: 10.3390/plants9121697 (PMC7761539; doi:10.3390/plants9121697)
Supplement: Supplementary file 1 [file plants-09-01697-s001.pdf]

plants

Table S1. Increment and reduction of CO_2 assimilation (An), stomatal conductance (gs) and intrinsic water-use efficiency (An/gs) between initial and final mean values (experimental time period) of stress treatment in seven bottle gourd landraces.

Landrace	Trait	Means values		Increment	Reduction
		Initial time	Final time	(%)	(%)
Osorno		17.16	2.6	-	84.8
Chépica		17.86	3.23	-	81.9
Illapel		10.3	1.96	-	81.0
Aurora	An	12.73	2.13	-	83.3
BG-48		14.03	7.73	-	44.9
BG-67		16.96	1.5	-	91.2
GC		18.76	6.23		66.8
Osorno		0.25	0.04	-	84.0
Chépica		0.25	0.04	-	84.0
Illapel		0.11	0.03	-	72.7
Aurora	gs	0.15	0.02	-	86.7
BG-48		0.17	0.06	-	64.7
BG-67		0.25	0.03	-	88.0
GC		0.27	0.05		81.5
Osorno		4.27	0.83	-	80.6
Chépica		4.43	0.87	-	80.4
Illapel		2.43	0.63	-	74.1
Aurora	E	2.97	0.57	-	81.0
BG-48		3.23	1.23	-	61.9
BG-67		4.53	0.70	-	84.5
GC		5.00	1.20	-	76.0
Osorno		73.3	57.3	-	21.8
Chépica		69.9	75	7.3	-
Illapel		90	58.4	-	35.1
Aurora	WUEi	86.7	76	-	12.3
BG-48		86.5	113.8	31.6	-
BG-67		67.2	45.8	-	31.8
GC		71.9	107.2	49.1	-

Table S2. Results of contrast test comparing the mean values difference between well-watered (WW) and water deficit (WD) condition for stomatal conductance (gs).

Genotypes	Estimate (WW-WD)	S.E[a]	p-value
Aurora	0.08	0.05	0.1221
BG-48	0.12	0.05	0.0215
BG-67	0.08	0.05	0.1267
Chepica	0.04	0.05	0.4780
GC	0.31	0.05	<.0001
Illapel	0.10	0.02	0.0439
Osorno	0.06	0.01	0.2056

[a]S.E= Standard Error.

Table 3. Results of contrast test comparing the mean values difference between well-watered (WW) and water deficit (WD) condition for transpiration (E).

Genotypes	Estimate (WW-WD)	S.E[a]	p-value
Aurora	1.17	0.55	0.0419
BG-48	1.70	0.55	0.0043
BG-67	1.43	0.55	0.0141
Chepica	0.67	0.55	0.2333
GC	3.23	0.55	<.0001
Illapel	1.77	0.55	0.0032
Osorno	1.00	0.55	0.0783

[a]S.E= Standard Error.

Table 4. Results of contrast test comparing the mean values difference between well-watered (WW) and water deficit (WD) condition for intrinsic water use efficiency (WUEi).

Genotypes	Estimate (WW-WD)	S.E[a]	p-value
Aurora	7.34	21.66	0.7374
BG-48	-28.87	21.66	0.1933
BG-67	23.40	21.66	0.2893
Chepica	23.97	21.66	0.2779
GC	-60.58	21.66	0.0092
Illapel	32.86	21.66	0.1405
Osorno	23.01	21.66	0.2971

[a]S.E= Standard Error.

Table 5. Results of contrast test comparing the mean values difference between well-watered (WW) and water deficit (WD) condition for instantaneous water use efficiency (WUEins).

Genotypes	Estimate (WW-WD)	S.E[a]	p-value
Aurora	0.87	1.14	0.4001
BG-48	-0.86	1.10	0.4073
BG-67	1.28	2.46	0.2191
Chepica	1.38	2.84	0.1872
GC	-1.86	5.17	0.0789
Illapel	2.34	8.19	0.0294

Osorno	1.35	2.72	0.1967

[a]S.E= Standard Error.

Table 6. Results of contrast test comparing the mean values difference between well-watered (WW) and water deficit (WD) condition for intercellular CO2 concentration (Ci).

Genotypes	Estimate (WW-WD)	S.E[a]	p-value
Aurora	-26.67	36.72	0.4738
BG-48	22.67	36.72	0.3055
BG-67	-49.33	36.72	0.1899
Chepica	-54.00	36.72	0.1526
GC	77.67	36.72	0.0435
Illapel	-80.67	36.72	0.0365
Osorno	-42.67	36.72	0.2551

[a]S.E= Standard Error.

Table 7. Results of contrast test comparing the mean values difference between well-watered (WW) and water deficit (WD) condition for CO_2 assimilation.

Genotypes	Estimate (WW-WD)	S.E[a]	p-value
Aurora	5.60	1.86	0.0055
BG-48	4.80	1.86	0.0155
BG-67	5.33	1.86	0.0078
Chepica	4.63	1.86	0.0190
GC	7.37	1.86	0.0005
Illapel	10.23	1.86	<0.0001
Osorno	4.17	1.86	0.0334

Table 8. Origin and geographical coordinates of seven bottle gourd genotypes evaluated under well-watered and water deficit conditions.

Genotype	Origin (country and region)	Geographical coordinates
BG-48	South Africa Kgohloane	(23 ° 47'39.76 " S; 29 ° 22'13.45 " E)
BG-67	South Africa Ga-Rapitsi	(23 ° 35'48.37 " S; 29 ° 06 '25 .08 " E)
GC (commercial)	South Africa Kwazulu-Natal	(29 ° 45’19.98’’ S; 29 ° 17'36.60 " E)
Illapel	Chile Coquimbo Region	(31 ° 35'28.58 " S; 70 ° 45'55.67 " O)
Chépica	Chile O'Higgins Region	(34 ° 23'50.69 " S; 71 ° 07'21.91 " O)
Osorno	Chile Los Lagos Region	(39 ° 22'31.44 " E; 72 ° 36'28.69 " OE)
Aurora	Commercial	NA
